# Face Damage Growth of Sandwich Composites under Compressive Loading: Experiments, Analytical and Finite Element Modeling

**DOI:** 10.3390/ma14195553

**Published:** 2021-09-24

**Authors:** Moustafa Kinawy, Felice Rubino, Giacomo Canale, Roberto Citarella, Richard Butler

**Affiliations:** 1 Independent Researcher, Derby DE23 6PT, UK; moustafa.kinawy@gmail.com; 2 Department of Chemical, Energetic and Mechanical Technology, University Rey Juan Carlos, Calle Tulipan, 28933 Mostoles, Spain; 3 Department of Mechanical and Aerospace Engineering, Bennett University, Plot Nos 8-11, TechZone II, Greater Noida 201310, India; c-giacomo.canale@bennett.edu.in; 4 Department of Industrial Engineering, University of Salerno, Via Giovanni Paolo II, 132-84084 Fisciano, Italy; rcitarella@unisa.it; 5 Materials & Structures Centre, Department of Mechanical Engineering, University of Bath, Claverton Down, Bath BA2 7AY, UK; r.butler@bath.a.uk

**Keywords:** composite sandwich, local buckling, critical delamination strain

## Abstract

Sandwich panels with composite laminate skins having [(±45_C_)_2_,(0_C_,0_G_)_4_,(±45_C_)_2_] stacking sequence (subscript C for carbon fibers, G for glass) and containing barely visible impact damage (BVID) induced on the whole sandwich structure impacted at low energy, were tested in edge after-impact-compression with load direction parallel and transversal to the fibers direction (0-dir.). The morphology of impact damage on the sandwich structure was determined by using ultrasonic C-Scan and visual observation of laminate cross section. A Digital Image Correlation (DIC) system was used to measure the delamination evolution during the test. Two different failure behaviors were observed in two different impacted panels. Panel with fibers oriented transversally to the compressive load showed an opening (Mode-I) propagation of a delamination, while the panel with fibers parallel to the load showed shear (Mode-II) propagation. The static load such to determine local buckling of the composite face and failure was experimentally measured. An analytical model was implemented to predict the static strength of laminate with Mode-I opening. An FE model was instead built to predict the local buckling failure mode of the laminate with BVID, which is the first phenomenon to appear. The results of the analytical model and the numerical simulation correlate well with the test.

## 1. Introduction

**Figure 1:**
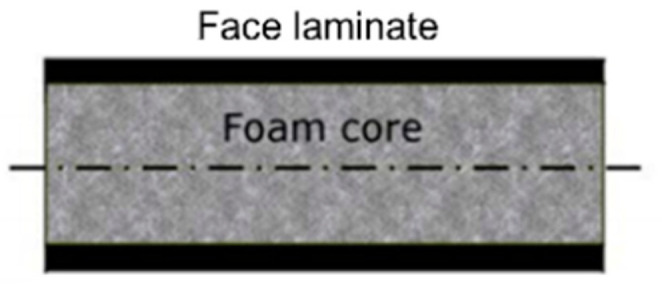
A scheme of a sandwich panel with an external face made by composite laminate and an inner “core”.

Sandwich composites are extensively used in many automotive, aerospace and marine industries due to high stiffness-to-weight ratios [1,2,3,4,5,6,7,8,9,10,11]. An example of sandwich structures is formed by two sub-components: an internal “core”, which resist the compression forces, and two external “faces”, or skin, that provide the flexural rigidity (see Figure 1).

As sandwich structures are subject to impact in many applications during their service life, many researchers in the last decades have investigated the influence of impact damage on static and fatigue strength and studied the damage mechanism of these structures. The authors of [11,12,13,14,15,16] experimentally investigated the compressive strength of impacted sandwich panels. They claim that the residual strength is 40% of the undamaged specimens. Kassapoglou et al. [17] developed an analytical model to predict compressive buckling of sandwich panels with elliptical cracks (delamination) in the area of the interface composite/core. Kassapoglou and Abbott used a simple buckling eigenvalue [18] to calculate the critical buckling load a local elliptical damage. Shipsha et al. [19] studied the effect of low velocity impact damage on residual compression and bending strength of sandwich plates. Other studies have also determined the post-impact strength of sandwich structures [20,21].

Local buckling due to delamination is known to be one of the major mechanisms for damage propagation in solid laminates and sandwich structures loaded in Compression After Impact (CAI). Various mathematical models and simulation tools have been proposed to evaluate the behavior of composite laminate under compressive loads and to represent delamination growth [22,23]. Chai et al. [24] developed a one-dimensional mathematical model of delamination propagation in composite plates with homogenized material properties. Chai and Babcock [25] developed a 2D local delamination-buckling model to study the sensitivity to the initial delamination size, the elastic properties and the loading history. Flanagan [26] developed a linear elastic fracture mechanics analytical model to predict growth of a two-dimensional damaged laminates subjected to compression. 

In this paper, the CAI strength of composite faces of sandwiches is investigated first experimentally and then by comparing the test results with the analytical model proposed in [27,28] and FE analysis. A Digital Image Correlation system (DIC) was used to understand the shape and size of the delamination with the test load progression. The results were compared with an analytical model [27,28,29] for prediction of CAI strength of composite laminates. The FE model was developed to predict local buckling, which is the first failure mode observed in the tests.

## 2. Materials and Methods

### 2.1. Experimental Setup

The damaged composite laminates (skin) of sandwich specimens previously impacted were tested under static loading. Before testing the damaged laminate in isolation, in fact, impact damage was induced on the entire sandwich. It was found that the damage induced by the low-speed impact concerns only one skin, i.e., one laminate. 

The Rohacell Polymethacrylamide Foam core was 25 mm thick. Material properties of the Core are given in Table 1.

The skin face materials were made by unidirectional glass and carbon fiber prepreg and epoxy matrix. Each skin is formed by a 2.2 mm thick composite laminate having sixteen layers. Material properties for the unidirectional layer are shown in Table 2 (in-plane) and Table 3 (out-of-plane). 

**Figure 2:**
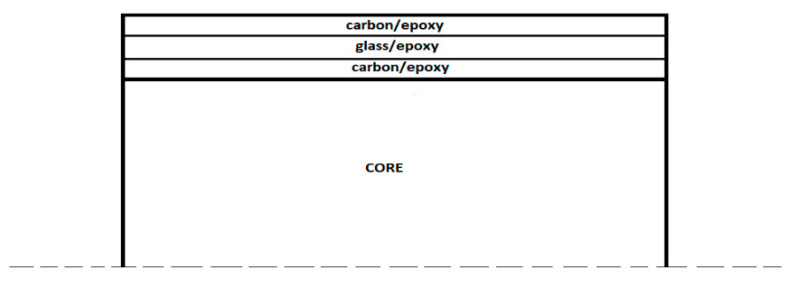
A scheme of the architecture of the tested sandwich panels (not on scale).

The properties of materials listed in Table 1 and Table 2 were provided by the manufactures and the other ones were taken from published literature [30,31]. The stacking sequence of the composite laminates was [(±45_C_)_2_,(0_C_,0_G_)_4_,(±45_C_)_2_], where sub-scripts *C* and *G* denote carbon and glass, respectively. The specimens had an original length of 220 mm, they were 100 mm wide and 29.4 mm thick (the core was 25 mm thick whilst the composite was 4.4 m thick, 2.2 mm each face). A scheme of the specimens is shown in Figure 2.

**Figure 3:**
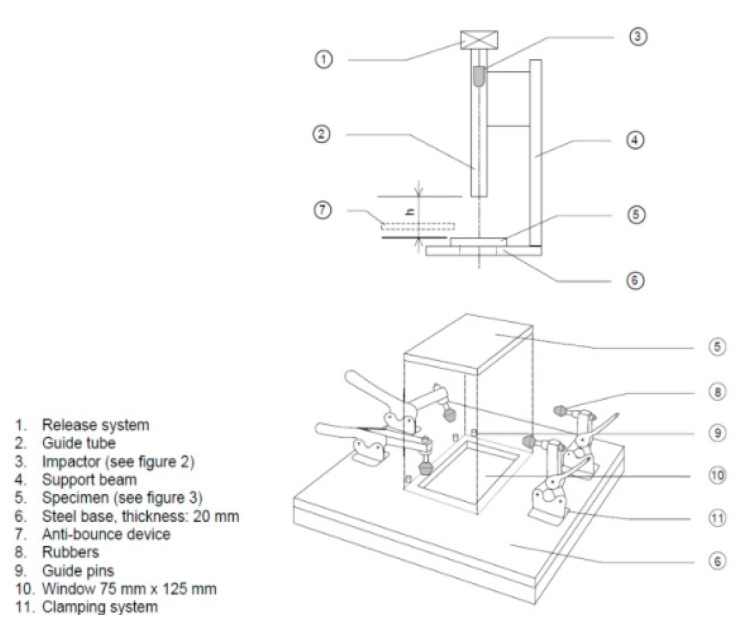
Test configuration for the low impact.

Two sandwich specimens were impacted by an 8J energy impactor by using an Instron/Dynatup impact machine (Instron, High Wycombe, Buckinghamshire, UK). They were held by a fixture which has an unsupported window (75 × 125 mm) directly under the impactor. The impactor head had a semi-spherical shape of 16 mm diameter. The (0°) fiber direction was aligned with the length of the window. The frame of the test machine configuration is given in Figure 3.

**Figure 4:**
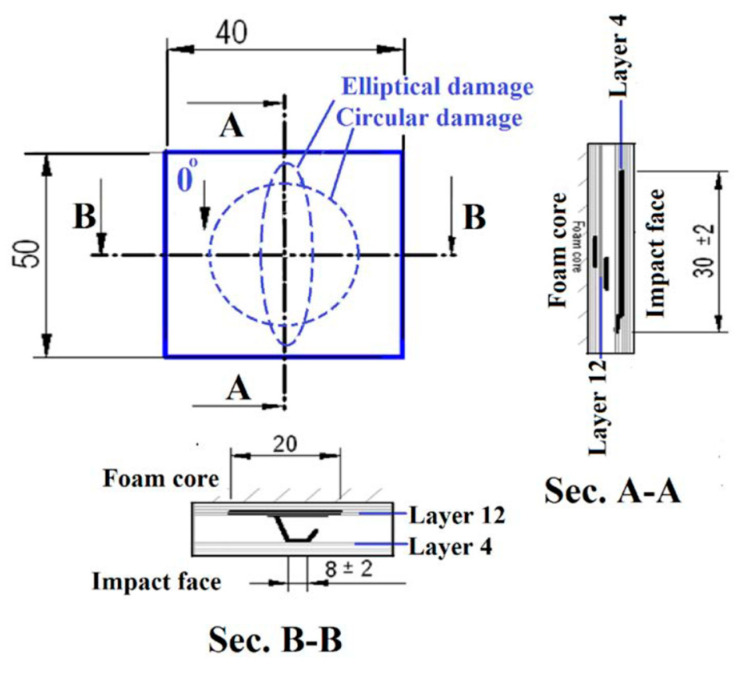
Sectional view of core specimen damage.

The impact-damaged specimens were scanned by using an Ultrasonic Sciences Ltd. C-Scan system (Ultrasonic Sciences Limited, Aldershot, United Kingdom) to understand the damage morphology caused by the impact on the laminate before the compression test. After the C-Scan, in order to obtain a better estimate of the damage, a cut-up was carried out at two perpendicular directions as presented in the scheme in the Figure 4 below.

#### Compression after Impact (CAI) Test

Two 100 mm × 75 mm specimens were tested in edge compression after the impact. The specimens were cut from the damage structures. The CAI fixture and specimen dimensions were designed to isolate the buckling of the composited sublaminate from other failure modes such as the global buckling, specimen end brooming or foam debonding. For this reason, a 2 mm space between the foam and the resin surface was ensured at each specimen side. This avoided any compression of the foam. Further, the specimen dimensions were selected so that no global buckling occurs during the load range. The specimens were rotated changing the orientation of the glass/epoxy layers with respect to the applied load to investigate the influence of impact on the static strength. The first specimen was aligned transversal to the loading axis (x-axis) so that it can be assumed having [(∓45_C_)_2_,(90_C_,90_G_)_4_,(∓45_C_)_2_] face layup, while the second was aligned parallel to the compression load, resulting in an actual [(±45_C_)_2_,(0_C_,0_G_)_4_,(±45_C_)_2_] stacking sequence of the composite external faces of the sandwich. From here on out, the specimens were labeled using the “actual” stacking sequence with respect to the direction of the compressive loads. Each of the two impacted specimens were tested in compression in two different directions (only the damaged laminates were tested).

The [(∓45_C_)_2_,(90_C_,90_G_)_4_,(∓45_C_)_2_] had the 38 × 10 elliptic post impact delamination between the 4th and the 5th ply whilst the [(±45_C_)_2_,(0_C_,0_G_)_4_,(±45_C_)_2_] specimen had a 30 × 30 circular delamination between the 12th and 13th ply.

**Figure 5:**
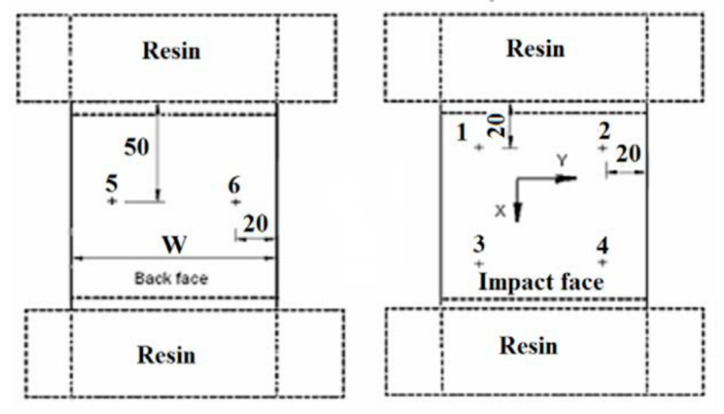
Sandwich specimen gauge length dimensions and strain gauge locations. Resin blocks were used for the [(±45_C_)_2_,(0_C_,0_G_)_4_,(±45_C_)_2_] face specimens.

In order to check the uniformity of the axial loading, six strain gauges were attached on the top and bottom faces of the laminates (Figure 5). 

Gauges 1, 2, 3 and 4 were applied on the top face. Gauges 5 and 6 were on the bottom face to ensure that the compression load was applied without inducing any bending. The widths W for the [(±45_C_)_2_,(0_C_,0_G_)_4_,(±45_C_)_2_] and [(∓45_C_)_2_,(90_C_,90_G_)_4_,(∓ 45_C_)_2_] laminates were 75 mm and 100 mm, respectively (the original impacted specimen was cut shorter for the compression after impact test). The dotted lines show the resin ends in which the specimens with [(±45_C_)_2_,(0_C_,0_G_)_4_,(±45_C_)_2_] face laminate are potted to prevent any brooming at the ends.

A Digital Image Correlation (DIC) system from LIMESS^®^ (LIMESS Messtechnik u. Software GmbH, Krefeld, Germany) was used to understand the damage evolution. Such a system was calibrated on the specimen at lower load level comparing the measurements with the data collected by the strain gauges. DIC measurements were conducted only during the after-impact compressive tests. 

### 2.2. Analytical Model

**Figure 6:**
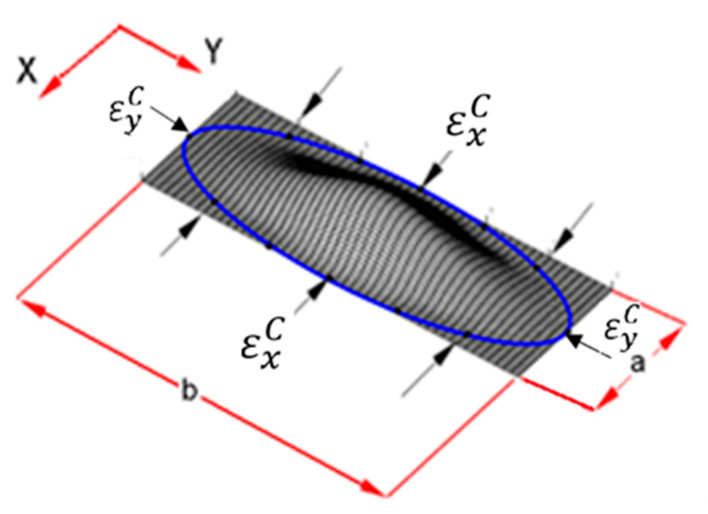
Thin-film model from [39] showing VICONOPT buckling modes for an elliptical sub-laminate.

The critical strain at which the local buckling occurs was calculated with an analytical model. This critical strain value was then used to calculate also the strain at which the delamination propagates [27,28]. Such an analytical model is the base of the well-established code VICONOPT [32]. In this software, the sub-laminate is represented as a series of finite strips, constrained by nodes approximating a circular or elliptic boundary, as shown in Figure 6. Along this circumference of ellipse, the thin sub-laminate is idealized as clamped. The critical strain depends on the composite stacking sequence, on the delamination size and shape and obviously on its position within the laminate. The threshold strain, at which the damage propagates, is calculated using a 2D analytical approach reported in [29]. Such a value, as already mentioned, uses the critical buckling strain estimated previously calculated by VICONOPT [33,34,35,36,37,38] as follows:

(1) $$\varepsilon_{th}=\varepsilon_{x}^{C}\left(\sqrt{4+\frac{G_{lC}}{u_{T}}}-1\right)$$

where $\varepsilon_{x}^{C}$, *G_lC_* and *u_T_* are respectively the critical buckling strain, the strain energy release rate for Mode-I propagation and the in-plane energy expressed in terms of principal buckling strains in the x and y directions: $\varepsilon_{x}^{C}$ and $\varepsilon_{y}^{C}$, respectively.

The initial damage morphology is the actual impact damage induced on the specimen. The two different damage morphologies of the two different CAI tests were modeled. 

### 2.3. Finite Element Model

**Figure 7:**
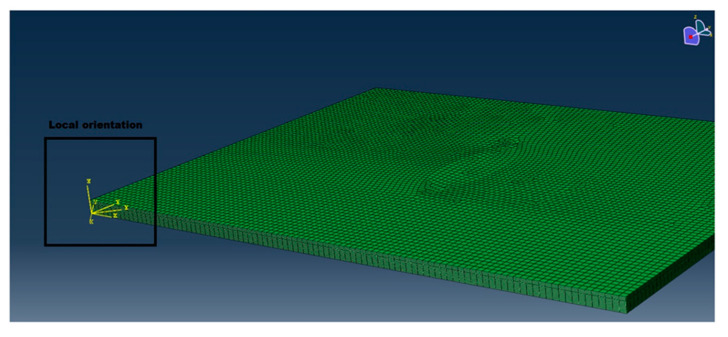
An overview of the mesh and of the local orientation of each ply.

Two different FE models were prepared by using Abaqus 6.14, the first one with an elliptic delamination (38 × 10) between the 4th and the 5th ply from the top surface, and the second with a circular delamination (30 × 30) between the 12th and 13th ply from the top according to the result from CT-scan analysis, showed in the subsequent paragraph. The dimensions of the panels are: 100 mm × 75 mm × 2.2 mm. The dimension adopted in the FE analysis are the same of those of the physical panel tested. A smaller damaged panel was in fact cut out from the sandwich specimen and then tested. HEX 20 elements were used for the mesh. The element was 1 mm long and two elements through the thickness were used for each ply. Each single ply were modeled with its own orientation according to the stacking sequence as shown in Figure 7.

**Figure 8:**
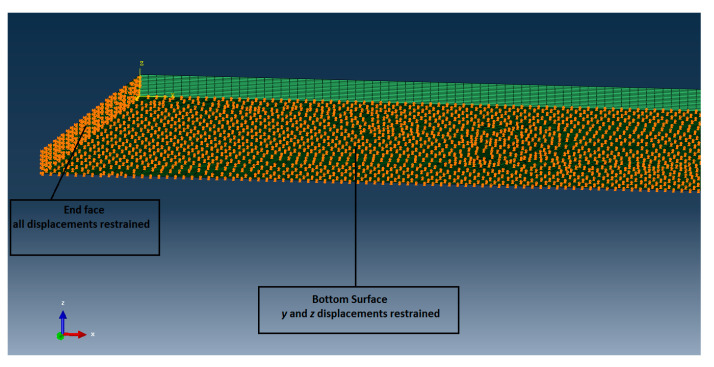
Boundary conditions of the two FE models.

Two sets of boundary conditions were applied. The surface opposite to the load application was fully restrained. The bottom surface of the laminate is free to move only along the x-axis, i.e., along the load direction. The boundary conditions are shown in Figure 8.

**Figure 9:**
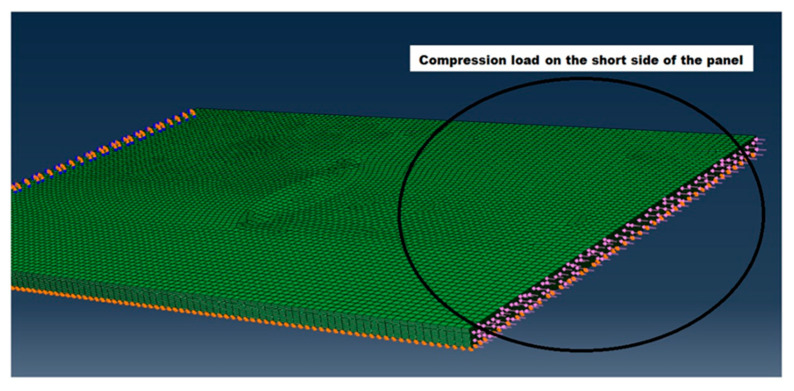
Compressive load applied.

A 1000 N compressive load was applied for the eigenvalue analysis in the form of a surface pressure as shown in Figure 9.

**Figure 10:**
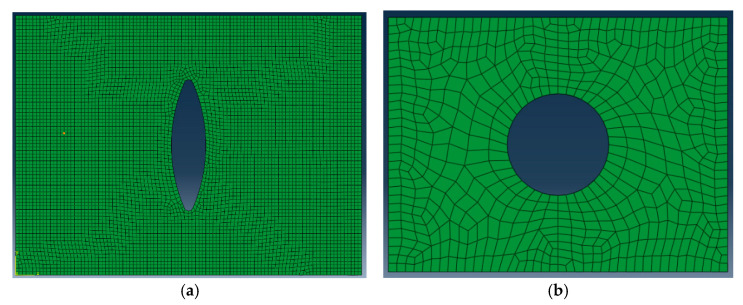
(**a**) The 38 × 10 delamination shape inserted between the 4th and 5th plies of specimen 1, with a 0.1 mm additional layer. (**b**) The 31 × 36 delamination shape inserted between the 12th and 13th plies of specimen 2, with a 0.1 mm additional layer.

The delamination was inserted by modeling an additional 0.1 mm layer containing the elliptical crack shape. The layer with the delamination for 38 × 10 mm elliptical crack model is shown in Figure 10a. The circular delamination 31 × 36 between the 12th and 13th ply is shown in Figure 10b. Mesh sensitivities studies were performed to make sure the mash density used in the analysis was not affecting the results.

## 3. Results

**Figure 11:**
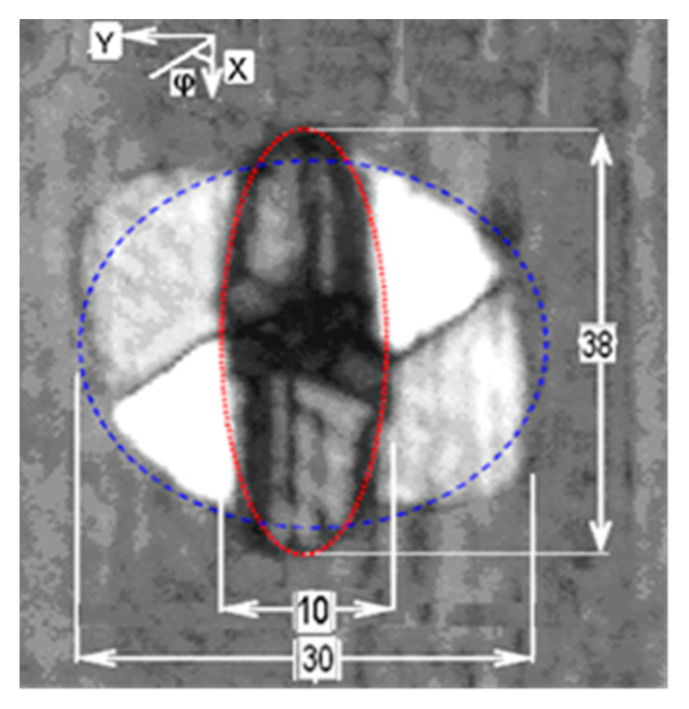
C-Scan image of the damage in a [(±45_C_)_2_,(0_C_,0_G_)4,(±45_C_)_2_] face laminate specimens. Dimensions are in mm and 0° plies are parallel to the x-axis of the image.

Two distinctive damage morphologies were observed in the C-Scan images of the tested laminates and after the cut-ups of 10 additional impacted sandwiches with same laminate definition that were not tested: one damage was of narrow elliptic shape (38 mm × 10 mm) near the impact surface, on the external thickness, while the other was of more regular circular shape (30 mm × 30 mm) near the core, but with the delamination, on the inner thickness of the composite face. The C-Scan image in Figure 11 shows the two different morphologies.

**Figure 12:**
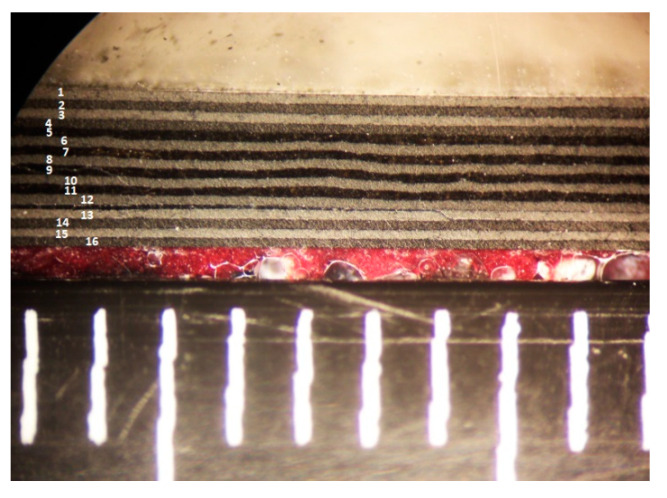
Cut up image shows separation between the 12th and the 13th layer.

To further investigate the through-thickness damage morphology, different samples (10 more) were impacted with the same energy value. The laminates were cut (only the first two were tested), and the cross-section examined and visualized with an optical microscope. A section at the center and along the x-axis (long side of the specimen) showed that the longest delamination of 38 mm length was located between the fourth and fifth ply starting from the external impact face of the sandwich. Another center section taken along the y-axis showed an average width of 8 mm at the same level and a 20 mm delamination between the 12th and 13th layers from the top (i.e., near the foam, on the inner thickness side), as show in Figure 12. 

Cross-section analysis agrees with the C-scan, despite a slight difference in the dimensions of the impact damage. After the cut-ups, it can be therefore concluded that the low speed impact has produced a damage of elliptical shape. The most likely locations for this laminate stacking sequence are likely to be at outermost plies of the block formed by eight consecutive 0-degrees plies. This is not an unexpected result. In industry, indeed, thick blocks with the same ply orientations are avoided just because their extremities could be a weak point because of the Poisson’s effect.

The occurrence of such defects is not determined: the defect in the sandwich structure analyzed after the impact could occur at 4/5th or 12/13th layers or simultaneously in the same laminate (as visible in Figure 10 and Figure 11), where there is the change in the orientation of the fibers. From the experimental evidence, on the other hand, it possible to argue that the shape of the defects at those locations is always the same, even though minimum variation in their size were observed. It means elliptical near the impact surface and circular deeper in the laminate: the darker the damage shape, the nearer it is to the surface.

**Figure 13:**
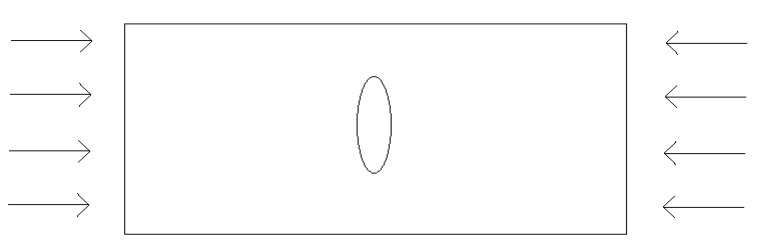
Original crack shape of [(∓45C)_2_,(90C,90G)_4_,(∓45C)_2_] specimen and load direction in CAI test.

**Figure 14:**
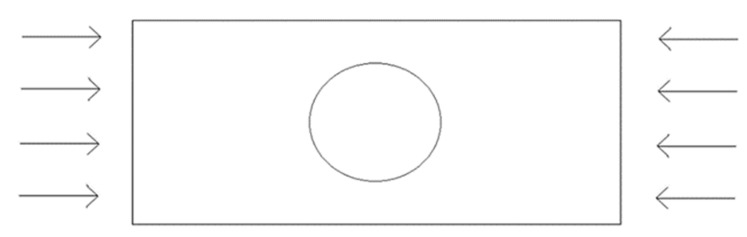
Original crack shape and compressive load for [(±45C)_2_,(0C,0G)_4_,(±45C)_2_] specimen.

The specimen showing the narrower delamination near the outer surface of the skin was tested with the fibers of the middle laminas oriented transversally to the compressive loads. i.e., the [(∓45_C_)_2_,(90_C_,90_G_)_4_,(∓45_C_)_2_] specimen. The impacted sample with the circular and deeper delamination was tested with fibers parallel to the load, i.e., [(±45C)_2_,(0C,0G)_4_,(±45C)_2_] specimen (see Figure 13 and Figure 14). 

**Figure 15:**
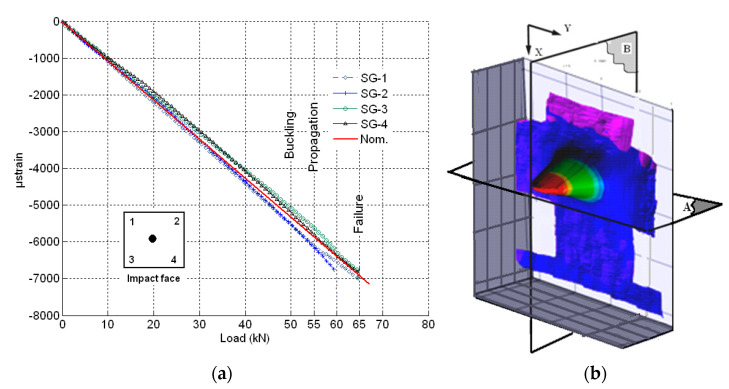
(**a**) Strain measurements for impacted face of the specimen with [(∓45_C_)_2_,(90_C_,90_G_)_4_,(∓45_C_)_2_] faces. (**b**) Out-of-plane displacements for specimen with [(∓45_C_)_2_,(90_C_,90_G_)_4_,(45_C_)_2_] faces at 65 kN (A represents the cutting plane parallel to y direction, B represents the cutting plane parallel to x direction).

The loading behavior of the [(∓45_C_)_2_,(90_C_,90_G_)_4_,(∓45_C_)_2_] specimen was linear up to failure at a load level of 65 kN as shown in Figure 15a. The failure was then a typical brittle failure of a composite material as the load carrying capability as a function of the compression displacement dropped abruptly.

**Figure 16:**
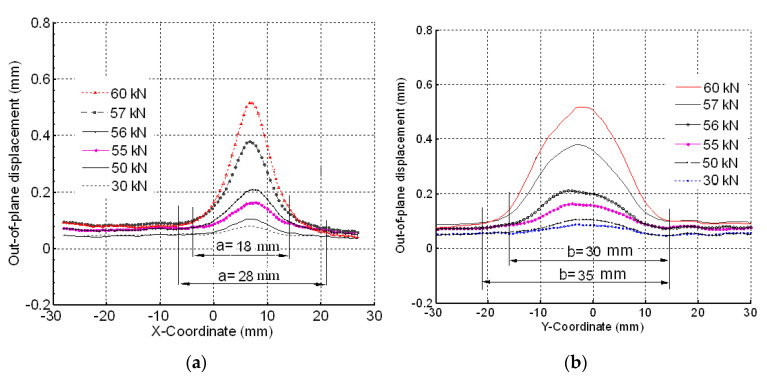
(**a**) Out-of-plane displacements seen when the vertical cut plane (B) of Figure 15b is used at different compression loads. (**b**) Out-of-plane displacements seen when horizontal cut plane (A) on Figure 15b is used at different compression load. (n.b. the curves were shifted slightly for clarity. In other words, the out-of-plane displacements were artificially increased to separate the curves from each other.)

A local buckling was found as shown in Figure 15b, in which out-of-plane displacements of the damaged laminate occurred at 65 kN. A measure of the out-of-plane displacements is reported in Figure 16.

The DIC results show that significant out of plane buckling displacement occurred at 48 kN and that it increased with the applied load. The DIC show also that the delamination started propagating above 55 kN. This is translated into a strain level of approximately 5820 µstrain. Nominal strain is obtained using the slope of the initial linear part of the load–strain curve to eliminate any bending influence. At the start of buckling, the damage had an elliptical base. The major axis of the ellipse was perpendicular to the loading axis (Figure 15b) and its vertical base dimension increased with increasing load; Figure 16a shows evidence of damage propagation. Noting that the curves in Figure 16a,b were plotted in groups of two load levels. These groups were slightly shifted vertically for clarity.

The maximum out-of-plane displacement was 0.7 mm measured at 65 kN corresponding to a nominal strain level of approximately 6880 µstrain.

In the case of the specimen with [(±45_C_)_2_,(0_C_,0_G_)_4_,(±45_C_)_2_] face laminates, whose initial crack had a quasi-circular shape (Figure 11), propagation occurred at a nominal strain level of 6950 μstrain. The subsequent failure occurred at 151 kN, which could be translated into a nominal strain of circa 7300 μstrain. 

**Figure 17:**
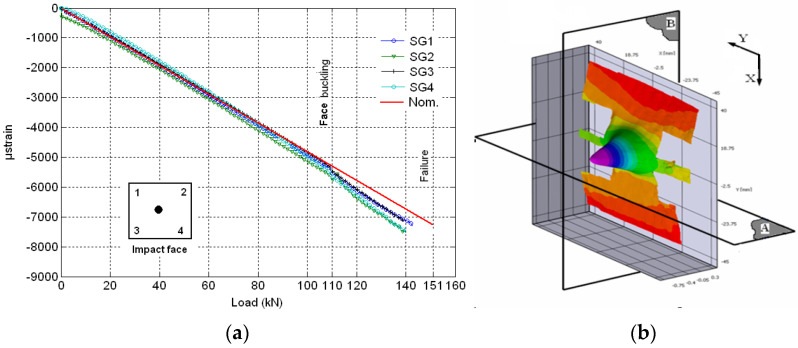
(**a**) Strain measurements for impact face of the specimen with [(±45_C_)_2_,(0_C_,0_G_)_4_,(±45_C_)_2_] faces. (**b**) Out-of-plane displacement for specimen with [(±45_C_)_2_,(0_C_,0_G_)4,(±45_C_)_2_] faces at 151 kN.

**Figure 18:**
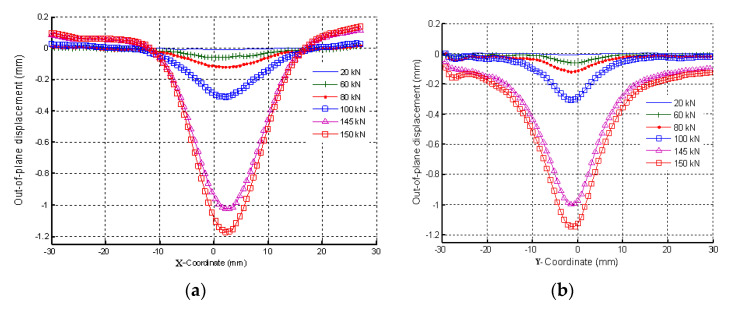
(**a**) Out-of-plane displacements on the cut plane (B) of Figure 10 at different compression loads. (**b**) Out-of-plane displacements on the cut plane (A) of Figure 10 at different compression loads (n.b. the curves were shifted slightly for clarity. In other words, the out-of-plane displacements were artificially increased to separate the curves from each other.)

In contrast to the previous specimen, global buckling was observed to occur towards the foam side (inwards). The strain measurements are given in Figure 17a while Figure 17b and Figure 18a,b show out-of-plane displacements. The displacement distribution had the shape of a cone. The base was a circle. The maximum out-of-plane displacement was 1.2 mm. The radius of the circular base was approximately 30 mm. Local buckling of the specimen was observed at 110 kN. The specimen failure was abrupt. Cracks were visible in most of the top surface.

The specimen with [(±45_C_)_2_,(0_C_,0_G_)_4_,(±45_C_)_2_] laminate has a high value of in-plane Poisson’s ratio (*ν_xy_* = 0.6). This implies a lower value of lateral compressive stress induced in the delaminated (±45_C_)_2_ sub-laminate. This retarded the buckling compared with the equivalent stress for the [(∓45_C_)_2_,(90_C_,90_G_)_4_,(∓45_C_)_2_] laminate, whose Poisson’s ratio is ca 0.3. 

**Figure 19:**
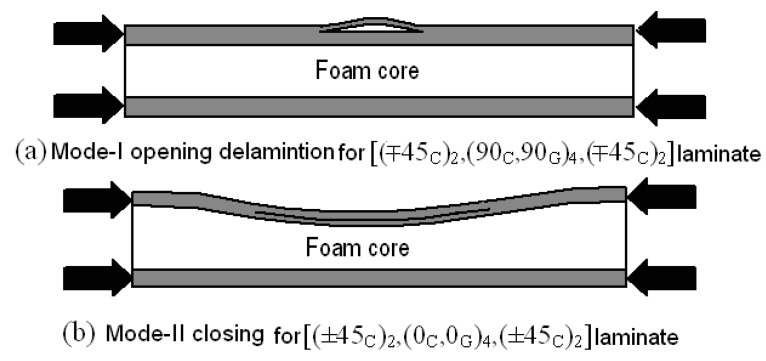
(**a**,**b**) Different buckling modes of the two sandwich specimens with different face laminates.

Devoted literature points out that the buckling shape of the skin after delamination can follow two preferred paths: it can open or close a crack like that shown in Figure 17 [29]. For what concerns the analytical model, the assumed buckling shape is the one in which a delamination is open (Figure 19a). A closing mode of the crack was also observed for some other laminates tested (see Figure 19b), but this is not reported or modeled here in this paper. 

The adopted analytical model [40] assumes that propagation is caused by Mode-I opening of a delamination at a critical interface within the laminate. Hence, the model was used to predict the buckling and threshold strain for the face laminate in which such opening occurred (this is not applicable when the local buckling presents the closure mode shown in Figure 17b). The C-Scan image in Figure 10 showed damage morphology through the face thickness while the sectioning technique showed the maximum delamination was between the fourth and fifth layers. DIC results taken for the [(∓45_C_)_2_,(90_C_,90_G_)_4_,(∓45_C_)_2_] specimen showed that the buckled damage area had an elliptic base of dimensions a = 18 mm and b = 30 mm at a load level of 55 kN after which unstable propagation took place. This size of delamination between the fourth and fifth layers was used in the model with a clamped–clamped condition assumed at each end of the sub-laminate. Failure of sandwich under the compressive load due to debonding of composite laminate face from the foam core was not considered in the present work. The impact crack is indeed localized within the laminate, as detected from the C-scan (see Figure 10); no traces of interface cracks were found that could propagate when compressive load is applied to the structure, leading to the face/core dis-bond [41,42,43,44]. This could be due specific features designed for the compressive tests that allowed to isolate the local buckling of the sublaminate face from the global buckling of the sandwich structure.

The analytical model assumes opening of the delamination like shown in Figure 17a. Since this does not occur for the [(±45_C_)_2_,(0_C_,0_G_)_4_,(±45_C_)_2_] laminate specimens, the propagation model is not applicable in that case. The predictions for the 90°-dominated laminate are compared with the experimental results in Table 4. 

From the compressive static test of the [(∓45_C_)_2_,(90_C_,90_G_)_4_,(∓45_C_)_2_] specimen, the buckled surface coming out of plane was of elliptic shape (Figure 15b). Propagation, which was predicted by the mathematical model [40] to occur at 6130 μstrain, was observed in the experiment at a nominal strain of approximately 5820 μstrain. Unstable propagation leading to failure did not occur until a nominal strain of 6880 μstrain. 

Although the experimental end conditions for the [(∓45_C_)_2_,(90_C_,90_G_)_4_,(∓45_C_)_2_] specimens and the [(±45_C_)_2_,(0_C_,0_G_)_4_,(±45_C_)_2_] specimens were different, failure occurred at the centrally located site of impact damage where the measured strains were consistent with the assumption of uni-axial load.

Hunt et al. [29] showed that the occurrence of an opening or closing mode depends on the location and length of the delamination. A long and narrow delamination is more likely to produce an opening mode because the local sub-laminate local buckling is observed before the global buckling of the whole panel. Isotropic material and full width delaminations were among the main assumptions of their work. The study reported in this paper, on the other hand, showed that a delamination depth to total face thickness ratio of 0.246 in the [(∓45_C_)_2_,(90_C_,90_G_)_4_,(∓45_C_)_2_] laminate resulted in an opening mode, while the same ratio in the [(±45_C_)_2_,(0_C_,0_G_)_4_,(±45_C_)_2_] laminate produced a closing mode. It can be claimed that the study presented here is in line with the findings of [32].

It is evident from the experimental campaign that the first failure, at least from the design point of view, was the local buckling for the plies above the delamination. Such a local buckling event was observed for both the panels with different elliptic shapes and it preceded the first sign of delamination. An attempt was made to reproduce the test results with the simplest possible FE simulation technique. It is, in fact, beyond the scope of this paper to treat the buckling problem of composite laminates, for which we point in the direction of other literature [45,46]. From the industrial point of view, the arc-length simulations, like Abaqus RIKS, have given very good results. The quickest evaluation is, however, given by the buckling eigenvalue analysis, and this is the strategy chosen by the authors to obtain results with the minimum computational effort.

**Figure 20:**
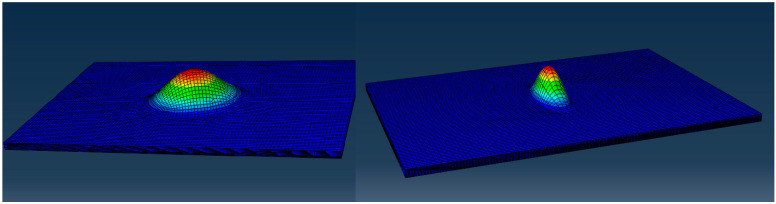
Eigen-solution results for the two different elliptical damage shapes, one (38 × 10) between the 4th and 5th ply and the other one (31 × 36) between the 12th and 13th ply.

The eigenvalue analysis was performed and the results are shown in Figure 20.

The predicted local buckling for the 0°dominated specimen with the 30 × 30 delamination is 114 kN. This value is difficult to correlate to the experiment as no buckling mode was observed experimentally. 

On the other hand, the predicted local buckling for the specimen with elliptical delamination was 44 KN. The FE buckling analysis under-estimated the critical buckling load measured during the test. An error of 8% was estimated for the 38 × 10 elliptical delamination. The cause of the discrepancy observed between numerical results and experiments could be ascribed to the boundary conditions; indeed, in the finite element method, blocking the displacements is a far too rigid assumption compared to reality. Another potential source of error could be the shape of the crack itself, being an idealization with a regular shape of dimensions not dissimilar to reality. Although the eigenvalue is the simplest FE procedure known by the authors for this type of estimation, the FE model is time consuming in the preprocessing phase and a non-negligible margin of error exists. It is therefore recommended to use the analytical model which has an error of 5% compared to the experimental buckling strain value. For this kind of problem, the analytical model proposed in [40] gives less than 3% error when compared to the experimental propagation strain results of the specimen with local delamination buckling. Such a simple FE analysis is however capable of giving an acceptable trend and is able to help the analyst gaining confidence on the physical phenomenon involved. The FE analysis, furthermore, was able to predict the shape of the buckled panel.

## 4. Conclusions

Two sandwich specimens with [(∓45_C_)_2_,(90_C_,90_G_)_4_,(∓45_C_)_2_] (specimen 1) and [(±45_C_)_2_,(0_C_,0_G_)_4_,(±45_C_)_2_] (specimen 2) face laminates were first impacted with a 8J hard impactor. Elliptical delamination was observed at the edges of the 8-plies oriented at 0 degrees. This was expected because different stiffness of this block of laminae compared to the ±45 blocks. Two different delamination shapes were found in two different impacted specimens. For specimen 1, the delamination was found between the 4th and the 5th ply, of dimension 38 × 10 mm. For specimen 2, the delamination was found between the 12th and the 13th ply, of an approximate dimension 30 × 30 mm. The delamination shapes of the defects found with the scans were confirmed by cut-ups on a third impacted specimen, not tested in CAI. The two damaged composite plates (without the core of the sandwich) were tested in compression after a low-speed impact damage was induced. The failure load was dependent on the fiber orientation at the center of the laminates and on the dimension and stacking sequence position of the damage. The 2D analytical tool was able to predict the local buckling and the propagation strain after impact with an error lower than 3%. A simple FE model was also built up for an approximate first level assessment of the local buckling. The error of the FE analysis was in any case below 10%. 

## Figures and Tables

**Table 1:** Core elastic properties.

Density [Mg/mm^3^]	Elastic Modulus [MPa]	Poisson’s Ratio
0.3 × 10^−9^	390	0.3

**Table 2:** In-plane material properties of unidirectional layers in sandwich faces under compression.

Fibers.	E11 (MPa)	E22 (MPa)	G12 (MPa)	ν12	G1C (J/m^2^)	Thickness (mm)
Fibredux 913 GE5—Unidirectional Glass	43,900	15,400	4290	0.28	225	0.142
Hexply 913C HTA Unidirectional Carbon	135,000	18,500	4970	0.29	225	0.134

**Table 3:** Out-of-plane material properties of unidirectional layers in sandwich faces under compression.

Fibers.	E33 (MPa)	G13 (MPa)	G23 (MPa)	ν13	V23
Fibredux 913 GE5—Unidirectional Glass	10,400	3000	3000	0.28	0.28
Hexply 913C HTA Unidirectional Carbon	10,400	3000	3000	0.29	0.29

**Table 4:** Summary of analytical and experimental results for buckling and threshold strains for specimens with [(∓45_C_)_2_,(90_C_,90_G_)_4_,(∓45_C_)_2_] face laminates, assuming that the delamination is after the fourth ply.

Sublaminate Dimensions	Analytical		Experimental	
a × b (mm) 18 × 30	ε^*C*^ (10^−6^)	ε_*th*_ (10^−6^)	ε^*C*^ (10^−6^)	ε_*prop*_ (10^−6^)
	5310	5970	5050	5820

## Data Availability

Data sharing not applicable.
